# HIV-1 subtype distribution and its demographic determinants in newly diagnosed patients in Europe suggest highly compartmentalized epidemics

**DOI:** 10.1186/1742-4690-10-7

**Published:** 2013-01-14

**Authors:** Ana B Abecasis, Annemarie MJ Wensing, Dimitris Paraskevis, Jurgen Vercauteren, Kristof Theys, David AMC Van de Vijver, Jan Albert, Birgitta Asjö, Claudia Balotta, Danail Beshkov, Ricardo J Camacho, Bonaventura Clotet, Cillian De Gascun, Algis Griskevicius, Zehava Grossman, Osamah Hamouda, Andrzej Horban, Tatjana Kolupajeva, Klaus Korn, Leon G Kostrikis, Claudia Kücherer, Kirsi Liitsola, Marek Linka, Claus Nielsen, Dan Otelea, Roger Paredes, Mario Poljak, Elisabeth Puchhammer-Stöckl, Jean-Claude Schmit, Anders Sönnerborg, Danika Stanekova, Maja Stanojevic, Daniel Struck, Charles AB Boucher, Anne-Mieke Vandamme

**Affiliations:** 1 Unidade de Saúde Pública Internacional e Bioestatística, Instituto de Higiene e Medicina Tropical, Universidade Nova de Lisboa, Lisboa, Portugal; 2 Centro de Malária e Outras Doenças Tropicais, Instituto de Higiene e Medicina Tropical, Universidade Nova de Lisboa, Lisboa, Portugal; 3 Department of Medical Microbiology, University Medical Center Utrecht, Utrecht, the Netherlands; 4 Department of Hygiene Epidemiology and Medical Statistics, Medical School, University of Athens, Athens, Greece; 5 Rega Institute for Medical Research, KU Leuven, Leuven, Belgium; 6 StatUa Center for Statistics, Universiteit Antwerpen, Antwerpen, Belgium; 7 Department of Virology, Erasmus Medical Center, Rotterdam, the Netherlands; 8 Department of Microbiology, Tumor and Cell Biology, Karolinska Institutet, Stockholm, Sweden; 9 Department of Clinical Microbiology, Karolinska University Hospital, Stockholm, Sweden; 10 Section for Microbiology and Immunology, The Gade Institute, University of Bergen, Bergen, Norway; 11 University of Milan, Milan, Italy; 12 Department of Virology, National Center of Infectious and Parasitic Diseases, Sofia, Bulgaria; 13 Hospital Egas Moniz, Centro Hospitalar de Lisboa Ocidental, Lisboa, Portugal; 14 irsiCaixa AIDS Research Institute & Lluita contra la SIDA Foundation, Hospital Universitari "Germans Tria si Pujol", Badalona, Spain; 15 University College Dublin, Dublin, Ireland; 16 National Public Health Surveillance Laboratory, Vilnius, Lithuania; 17 Sheba Medical Center, Ramat Gan, and Tel Aviv University, Tel Aviv, Israel; 18 Robert-Koch Institute, Berlin, Germany; 19 Warsaw Medical University and Hospital of Infectious Diseases, Warsaw, Poland; 20 Infectology Center of Latvia, Riga, Latvia; 21 Institut für Klinische und Molekulare Virologie, University of Erlangen-Nuremberg, Erlangen, Germany; 22 University of Cyprus, Nicosia, Cyprus; 23 National Institute for Health and Welfare, Helsinki, Finland; 24 National Institute of Public Health, Prague, Czech Republic; 25 Statens Serum Institute, Copenhagen, Denmark; 26 Molecular Diagnostics, "Prof. Dr .Matei Bals" Institute for Infectious Diseases, Bucharest, Romania; 27 University of Ljubljana, Ljubljana, Slovenia; 28 Medical University Vienna, Vienna, Austria; 29 Laboratory of Retrovirology, CRP-Santé, Luxembourg, Luxembourg; 30 Centre Hospitalier de Luxembourg, Rollengergronn-Belair-Nord, Luxembourg; 31 Divisions of Infectious Diseases and Clinical Virology, Karolinska Institute, Stockholm, Sweden; 32 Slovak Medical University, Bratislava, Slovakia; 33 University of Belgrade, School of Medicine, Belgrade, Serbia

## Abstract

**Background:**

Understanding HIV-1 subtype distribution and epidemiology can assist preventive measures and clinical decisions. Sequence variation may affect antiviral drug resistance development, disease progression, evolutionary rates and transmission routes.

**Results:**

We investigated the subtype distribution of HIV-1 in Europe and Israel in a representative sample of patients diagnosed between 2002 and 2005 and related it to the demographic data available. 2793 PRO-RT sequences were subtyped either with the REGA Subtyping tool or by a manual procedure that included phylogenetic tree and recombination analysis. The most prevalent subtypes/CRFs in our dataset were subtype B (66.1%), followed by sub-subtype A1 (6.9%), subtype C (6.8%) and CRF02_AG (4.7%). Substantial differences in the proportion of new diagnoses with distinct subtypes were found between European countries: the lowest proportion of subtype B was found in Israel (27.9%) and Portugal (39.2%), while the highest was observed in Poland (96.2%) and Slovenia (93.6%). Other subtypes were significantly more diagnosed in immigrant populations. Subtype B was significantly more diagnosed in men than in women and in MSM > IDUs > heterosexuals. Furthermore, the subtype distribution according to continent of origin of the patients suggests they acquired their infection there or in Europe from compatriots.

**Conclusions:**

The association of subtype with demographic parameters suggests highly compartmentalized epidemics, determined by social and behavioural characteristics of the patients.

## Background

Human immunodeficiency virus type 1 (HIV-1) is characterized by extensive genetic diversity. HIV-1 strains are divided in four groups (M, N, O and P), originating from four separate cross-species transmissions from chimpanzees and/or gorillas to humans. While HIV-1 groups O, N and P are mainly restricted to Central Africa, group M has caused the HIV pandemic [1-4]. HIV-1 group M has been further classified into 9 distinct subtypes, sub-subtypes and inter-subtype circulating recombinant forms (CRFs). Subtypes and sub-subtypes arose from founder effects at different time points in the past, and inter-subtype recombinants can arise in patients co-infected with strains from two different subtypes. If these newly recombined strains have a significant epidemic spread, they are called Circulating Recombinant Forms (CRFs) [5].

The spread of HIV-1 subtypes is important for epidemiological purposes but can also be of relevance in clinical settings. Some biological properties differ between subtypes. They have different rates of evolution and their sequence variation may affect antiviral drug resistance development [6-12], but overall limited differences are found in the genetic barrier to drug resistance development between subtypes [13]. Other studies suggested differences in disease progression: subtype D seems to have a faster disease progression than subtypes A or C [14,15]. In the absence of antiretroviral prophylaxis, subtype C is transmitted from mother-to-child more frequently compared to subtype D, which in turn is more frequently transmitted than subtype A [16,17]. Some studies suggest that sexual transmission of subtype C is also more likely than of subtypes A and D [18,19]. In addition, it is still not well understood how to cope with the genetic variability of HIV-1 for the development of an efficient HIV-1 vaccine [20-22].

Hemelaar *et al.* documented the molecular epidemiology of HIV-1 in the world in 2011 using convenience sampling and a literature review. Subtype C was described as the most prevalent globally, representing 48% of the infections, while subtypes A, B, CRF02_AG, CRF01_AE, subtype G and D accounted for 12, 11, 8, 5, 5 and 2% of the infections, respectively. In this study, subtype B accounted for 85% of HIV-1 infections in Western and Central Europe, while subtype A, C and G followed, with 2-3% of infections [23]. Another manuscript by the EuroSIDA study group also based on analysis of HIV-1 genomic sequences from 939 HIV-1 patients from Europe, Israel and Argentina followed from May 1994 onwards, documented a subtype B prevalence of 86%, 2% of subtype A, 4% of subtype C and 7% of other subtypes [24].

We had access to sequences from the SPREAD (Strategy to Control Spread of HIV Drug Resistance) surveillance programme, which is coordinated by the European Society for Antiviral Resistance (ESAR). This programme was initiated with the objective of reliably determining the prevalence of transmission of drug resistance within the different patient risk-groups and to identify risk factors enhancing the risk of transmission of drug resistance. A second objective was to characterize the epidemiological and sequence diversity of HIV-1 in Europe. Different than in previous approaches, in this study the samples were collected in a representative way from newly diagnosed patients (http://www.esar-society.eu/). In this paper, we describe the subtype distribution of HIV-1 in Europe and Israel, based on the SPREAD sequences of three collection periods from patients newly diagnosed between 2002 and 2005 [25,26].

## Results

### Subtype B accounts for 70% of HIV-1 infections in newly diagnosed patients living in Europe

Of the 2730 sequences included in the study, 2469 (90.4%) were successfully subtyped using the REGA Subtyping Tool version 2, while 261 (9.6%) were unclassified, of which 137 sequences (5.0%) remained untypable even after manual analysis. The subtypes with the highest proportion of new diagnoses were subtype B - 66.12% [64.3-67.9%], sub-subtype A1 - 6.9 [6.0-7.9%], subtype C - 6.8% [5.9-7.8%] and subtype G - 3.8% [3.1-4.6%]. Among the recombinants, the most common CRFs were: CRF02_AG – 4.7% [4.0-5.6%] and CRF01_AE – 4.0% [3.3-4.8%]. The proportion of U/URFs in this dataset was 5.0% [4.2-5.9%] (Table 1). When adjusting for oversampling in some countries (Additional file 1: Figure S1), the proportion of new diagnoses with subtype B increased to 70.2%; subtypes C and A decreased to 5.0 and 3.6% respectively; CRF02_AG and subtype G increased to 4.9% and 4.8% respectively; CRF01_AE decreased to 1.9%; and U/URFs increased to 5.8% (Additional file 2: Figure S2). Even though some of these differences are statistically significant, they are limited to the extent that they have no substantial impact on the remaining analyses, and are thus not further reported separately. All adjusted analyses can be found in supplementary materials.

**Table 1:** Percent subtypes for the complete set of patients and only for patients originating from SPREAD countries and 95% confidence intervals

	**All patients**	**95% C.I.**	**Patients originating from SPREAD countries**	**95% C.I.**	**p-value**
A1	**6.92**	[6.00-7.94]	**5.24**	[4.30-6.32]	p=0.012
B	**66.12**	[64.31-67.90]	**79.48**	[77.63-81.25]	p<2.2x10^-06^
C	**6.78**	[5.86-7.79]	**2.55**	[1.90-3.34]	2.4x10^-11^
D	**0.81**	[0.51-1.22]	**0.15**	[0.03-0.45]	N.S.
F	**1.28**	[0.89-1.78]	**1.12**	[0.70-1.69]	N.S.
G	**3.81**	[3.12-4.60]	**3.31**	[2.56-4.20]	N.S.
AE	**3.95**	[3.26-.76]	**2.60**	[1.94-3.40]	p=0.009
AG	**4.73**	[3.96-5.59]	**1.73**	[1.20-2.411]	p=2.1x10^-08^
Others	**0.59**	[0.34-0.95]	**0.20**	[0.06-0.52]	N.S.
U/URFs	**5.02**	[4.23-5.91]	**3.62**	[2.83-4.54]	N.S.

P-values are for comparisons between the complete dataset and for the dataset including only patients originating from SPREAD countries.

AE – CRF01_AE.

AG – CRF02_AG.

Others – Samples classified as other subtypes or CRFs.

A1 – Sub-subtype A1.

B – Subtype B.

C – Subtype C.

D – Subtype D.

F – Subtype F.

G – Subtype G.

U/URFs – Samples classified as Untypable (U), partially Untypable or as Unique Recombinant Forms (URFs).

### HIV-1 molecular epidemiology is highly heterogeneous between European countries

**Figure 1:**
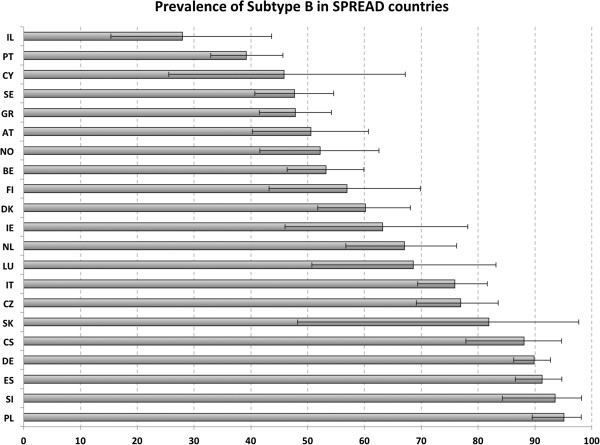
**Proportion of diagnoses with subtype B by country of sampling of the patient.** AT – Austria (n=99), BE – Belgium (n=220), CY – Cyprus (n=24), DK – Denmark (148), FI – Finland (n=48), DE – Germany (n=364), GR – Greece (251), IE – Ireland (n=38), IT – Italy (199), LU – Luxembourg (n=35), NL – Netherlands (n=97), NO – Norway (n=94), PL – Poland (n=121), PT – Portugal (n=240), SI – Slovenia (n=62), ES – Spain (n=206), SE – Sweden (n=210), CS – Serbia (n=67), CZ – Czech Republic (n=143), SK – Slovakia (n=11), IL – Israel (n=43).

The country of sampling corresponds to the country where the sample and questionnaire were collected. For most cases the “country of sampling” corresponds also to the area where the patient resides and is clinically followed. For all countries, the subtype with the highest proportion of new diagnoses was subtype B, except Israel, where subtype C was more prevalent than subtype B (58.1 vs 27.9%). The countries with the highest proportion of non-B subtypes were Israel and Portugal (72.1 % and 60.8%, respectively). This is due to the parallel epidemics of subtypes B and C and subtypes B and G in Israel and Portugal, respectively. Poland and Slovenia were on the opposite side, with the lowest proportion of non-B subtypes (5.0% and 6.5%) (Figure 1; Additional file 3: Figure S3 and Additional file 4: Table S1).

### New diagnoses with subtype B occurred in 79% of patients originating from and living in Europe

The country of origin corresponds to the country where the patient was born. When re-analyzing the distribution of subtypes including only patients who originated from SPREAD countries (n = 2225), the proportion of newly diagnosed patients infected with subtype B increased significantly from 66.1% to 79.5%. Subtypes or CRFs A1, CRF01_AE, CRF02_AG and C decreased significantly from 6.9%, 4.0%, 4.7% and 6.8% to 5.2%, 2.6%, 1.7% and 2.5% respectively, while the proportion of subtype G remained approximately stable (3.8% to 3.3%) (Table 1). A significant rise in proportion of newly diagnosed in this analysis means that the respective subtype is less found among the immigrant population.

### The proportion of new diagnoses with different subtypes among native populations is country-specific

Table 2 shows per country the 1^st^, 2^nd ^and 3^rd ^most prevalent subtypes sampled, as well as the 1^st^, 2^nd ^and 3^rd ^more prevalent subtypes among the native population. No consistent pattern exists for the proportion of new diagnoses with non-B subtypes among natives. In Belgium, the second most prevalent subtype was CRF02_AG in patients sampled in Belgium, while subtype C was more prevalent in patients originating from Belgium, implying that the high proportion of CRF02_AG is mostly caused by immigrants (in this case originating from Africa), while subtype C seems to have established among the Belgian population, alongside subtype B. In Portugal, subtype G is well established in the native population, while in Greece and Cyprus subtype A1 is well established in natives.

**Table 2:** **Proportion of new diagnoses with the 1**^**st**^**, 2**^**nd **^**and 3**^**rd **^**most observed subtypes in each country of sampling compared to 1**^**st**^**, 2**^**nd **^**and 3**^**rd **^**most observed subtypes among natives (unadjusted values only)**

	**1st**	%*	**2nd**	**%**	**3rd**	**%**
**AT samp**	B	50.50	CRF01_AE	13.13	AG	12.12
**AT origin**	B	61.76	CRF01_AE	16.18	A1	11.76
**BE samp**	B	53.18	CRF02_AG	10.45	C	8.18
**BE origin**	B	81.36*	C	5.93	CRF02_AG	2.54
**CY samp**	B	45.83	A1	37.50	C	8.33
**CY origin**	B	73.68	A1	21.05	C	5.26
**CZ samp**	B	76.92	A1	9.09	CRF01_AE	6.99
**CZ origin**	B	90.18*	A1	3.57	C	2.68
**DE samp**	B	89.84	CRF02_AG	3.30	CRF01_AE	2.20
**DE origin**	B	95.86*	CRF01_AE	1.59	CRF02_AG	0.96
**DK samp**	B	60.14	C	10.81	CRF01_AE and A1	7.43
**DK origin**	B	79.17*	AE and G	5.21	C	4.17
**ES samp**	B	91.26	CRF02_AG	1.94	G	0.97
**ES origin**	B	94.23	A1, C, F, G, AE, AG	0.64	-	-
**FI samp**	B	56.90	CRF01_AE	18.97	C	10.34
**FI origin**	B	59.65	CRF01_AE	19.30	C	10.53
**GR samp**	B	47.81	A1	29.88	C	5.98
**GR origin**	B	51.67	A1	33.01	C	3.34
**IE samp**	B	63.16	C	21.05	CRF02_AG	7.89
**IE origin**	B	91.30*	C	8.70	-	-
**IL samp**	C	58.14	B	27.91	A1	11.63
**IL origin**	B	100.00	-	-	-	-
**IT samp**	B	75.88	F	8.54	CRF02_AG	6.03
**IT origin**	B	85.43*	F	9.93	AG and A1	1.32
**LU samp**	B	68.57	C	11.43	G and U/URFs	8.57
**LU origin**	B	90.00	-	-	-	-
**NL samp**	B	67.01	CRF02_AG and C	9.28	A1	7.22
**NL origin**	B	88.00*	C and AG	4.00	-	-
**NO samp**	B	52.13	C	19.15	CRF01_AE	6.38
**NO origin**	B	86.27*	C	7.84	CRF02_AG	1.96
**PL samp**	B	95.04	CRF01_AE and C and F	0.83	-	-
**PL origin**	B	93.80	CRF01_AE, A1, C and F	0.77	-	-
**PT samp**	B	39.17	G	30.00	C	6.25
**PT origin**	B	47.75	G	30.33	CRF02_AG	2.81
**SE samp**	B	47.62	CRF01_AE and C	14.28	CRF02_AG	8.10
**SE origin**	B	73.47*	CRF01_AE	12.24	CRF02_AG	5.10
**SI samp**	B	93.55	CRF01_AE and CRF02_AG and A1	1.61	-	-
**SI origin**	B	96.61	CRF02_AG	1.69	-	-
**SK samp**	B	81.82	C	9.09	-	-
**SK origin**	B	87.50	C	6.25	-	-
**CS samp**	B	88.06	G	5.97	CRF01_AE and C	2.99
**CS origin**	B	88.20	G	5.88	CRF01_AE	2.38

* indicates that the difference between these two is significant. U/URFs were excluded from this table, since they represent a heterogeneous group of strains.

The most extreme case of discrepancy in infecting subtypes between natives and immigrants is Israel, where natives are exclusively infected with subtype B and the majority of infected immigrants - mainly from Ethiopia – are infected with subtype C.

### The HIV-1 subtypes infecting immigrant patients living in Europe are mostly similar to the HIV-1 subtypes causing epidemics in their country/continent of origin

When analyzing the distribution of subtypes of the patients originating from countries other than SPREAD countries, we found results consistent with our current HIV-1 molecular epidemiological knowledge in those countries, suggesting that the country where the patient originates is also the country where the infection was acquired or that they acquired their infection in Europe from compatriots (Additional file 5: Figure S4; Additional file 4: Table S2). The logistic regression results were consistent with these results by indicating continent of origin and countries of origin as significantly associated with the proportion of new diagnoses with different subtypes (see Additional file 4: Table S3 for details) while country of sampling was rarely associated with it.

**Figure 2:**
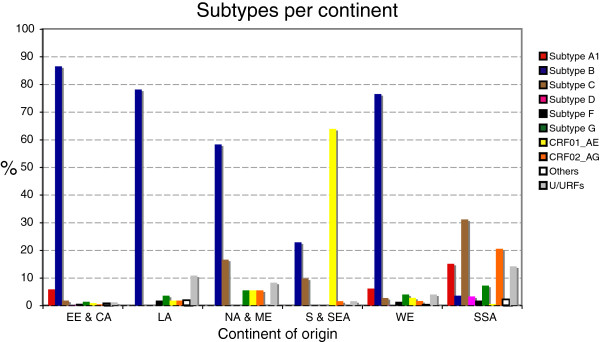
**Subtype distribution stratified by continent of origin of the patient.** Regional distribution of the countries was defined as in the UNAIDS reports (Hemelaar, et al., 2006): Sub-Saharan Africa (S-S A, n=330), East Asia (n=2), Oceania (n=1), South and South-East Asia (S/S-E A, n=61), Eastern Europe and Central Asia (EE/CA, n=425), Western Europe (WE, n=1636), North Africa and Middle East (NA/ME, n=36), North America (n=7), Caribbean (n=11), Latin America (LA, n=55). Due to the low sample size, East Asia and Pacific, Oceania, North America and Caribbean are not included in the figure. See Additional file 5: Table S5 for list of countries included in each continent region.

The distribution of subtypes according to the continent of origin of the patient is represented in Figure 2. The proportion of newly diagnosed with subtype B was higher in patients from Western Europe (76.59%), Latin America (78.18%) and Eastern Europe and Central Asia (86.59%). In patients from South and South-East Asia, the most prevalent subtype was CRF01_AE (63.93%). Finally, in patients from Sub-Saharan Africa, almost all subtypes were found, but subtype C seems to dominate the epidemic (31.21%). Albeit subtype B was the most prevalent subtype in patients originating from North Africa and Middle East (58.33%), subtype C was also very prevalent in these patients (16.67%), (Figure 2).

### HIV-1 molecular epidemiology in Europe is highly stratified according to gender and risk group

**Figure 3:**
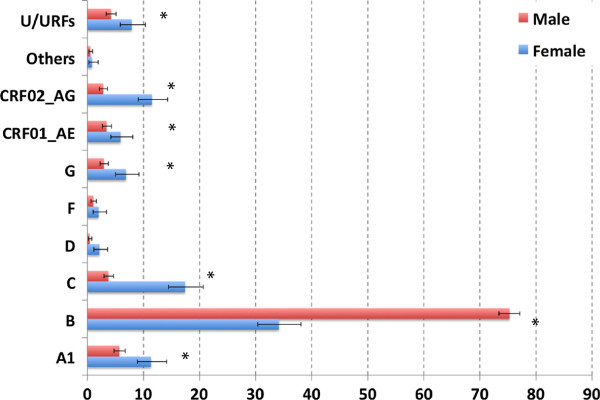
**Subtype distribution by gender.** A total of 307 females (blue bars) and 989 males (red bars) were included in the analysis. For 4 patients, this information was not available; and these were deleted from the data set. Asterisks indicate statistically significant differences in the proportion of a certain subtype in male vs. female (p<0.05).

The distribution of different subtypes according to gender is represented in Figure 3. The proportion of subtype B is significantly higher in men than in women, while the proportion of subtypes A1, C, G, CRF01_AE, CRF02_AG and U/URFs is significantly higher in women.

**Figure 4:**
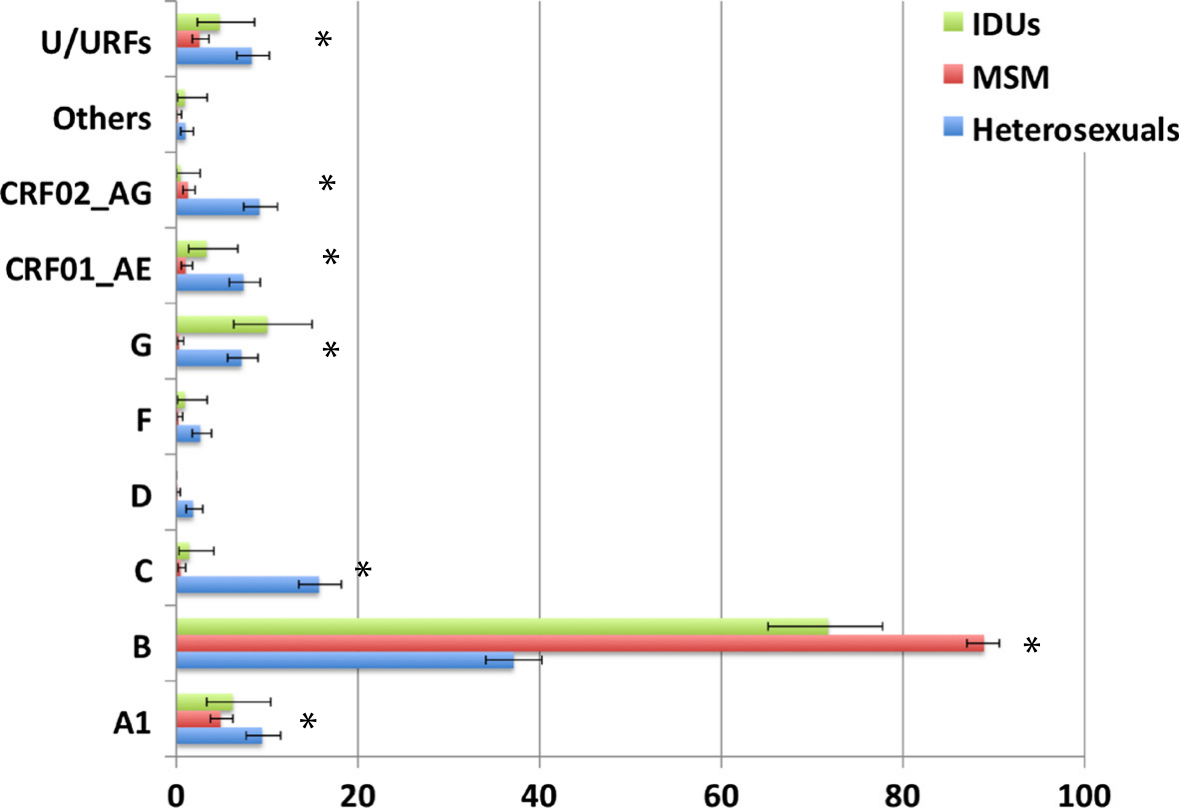
**Proportion of diagnoses with different HIV-1 subtypes in different risk groups.** IDUs (green bars) – intravenous drug users (n=221); Homo-bi (red bars) – homobisexuals (n=1271); Hetero (blue bars)– heterosexuals (n=994). Asterisks indicate significant differences in the proportion of one subtype between at least two of the risk groups. For example, for URFs the significant difference was found only between homosexuals and heterosexuals (p=1.2x10-5) while subtype B has a significantly different frequency in all risk groups. For more details, please refer to the methods and results sections.

The proportion of subtype B is significantly higher in MSM (men who have sex with men) patients than in IDUs and in heterosexuals and is significantly higher in IDUs than in heterosexual patients. Subtype A1 was significantly more prevalent in heterosexuals (9.48%) than in MSM (4.9%), but it was the 2^nd ^most prevalent subtype in MSM, with all other subtypes less than 1.3%. These patients were mostly from Greece (n=53), but also from Cyprus (n=3), Portugal (n=2), Spain (n=1), the Netherlands (n=1) and Ireland (n=1). The increase in proportion of subtypes A1 and C has also been described in the MSM population of the United Kingdom [27]. Subtype G was significantly less prevalent in MSM (0.32%) than in IDUs (10.05%) and heterosexuals (7.20%), while subtypes C, CRF02_AG and CRF01_AE were significantly more prevalent in heterosexuals (15.7%, 7.41% and 9.16% respectively) than in IDUs (1.44%, 3.35% and 0.48% respectively) and MSM(0.48%, 1.04% and 1.28% respectively). URFs were found significantly more in heterosexuals than in MSM (Figure 4).

Logistic regression indicated risk group MSM as a positive predictor of infection by subtype B, while heterosexual risk group is linked to infection with sub-subtype A1 and subtype C. Risk group MSM also indicates lower risk of infection with CRF01_AE, CRF02_AG and subtypes C and F. See Additional file 4: Table S3 for Odds Ratios.

### Continent and country of origin and risk factor are the main determinants of subtype distribution

Multinomial logistic regression indicated that gender, risk group and continent of origin were the main determinants of subtype distribution. Country of sampling was also indicated as a determinant of subtype distribution by the regression model, but the p-value (p>0.05) for this association was not significant (Additional file 4: Table S4).

**Figure 5:**
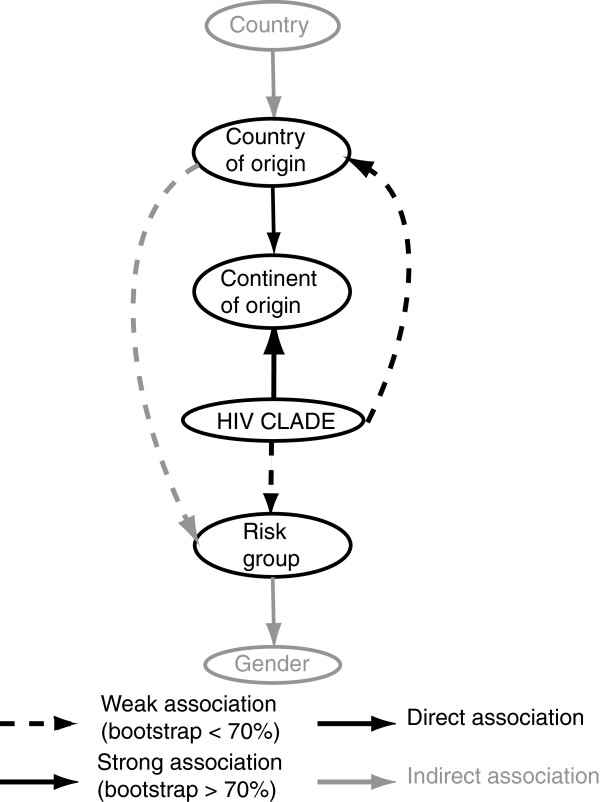
**Bayesian networks of the variables that were found to be associated with the subtype of the HIV-1 strain in the univariate analysis.** The variables gender, risk group and continent of origin were grouped as described previously. Black arcs indicate variables directly associated with the Subtypes/CRFs variable. Grey arcs indicate indirect association with Subtypes/CRFs. Dotted arcs indicate associations with low bootstrap support (<70%), while full arcs indicate associations confirmed by a high bootstrap support (>70%). Country of sampling included 20 countries: Austria, Belgium, Cyprus, Denmark, Finland, Germany, Greece, Ireland, Italy, Luxembourg, Netherlands, Norway, Poland, Portugal, Slovenia, Spain, Sweden, Serbia, Czech Republic, Slovakia and Israel. Country of origin included: Netherland Antilles, Angola, Argentina, Austria, Belgium, Burkina Faso, Burundi, Benin, Brasil, Democratic Republic of Congo, Congo, Switzerland, Cote D’Ivoire, Chile, Cameroon, China, Colombia, Cuba, Cape Verde, Cyprus, Czech Republic, Germany, Djibouti, Denmark, Dominican Republic, Algeria, Equator, Estonia, Egypt, Eritrea, Spain, Ethiopia, Finland, France, United Kingdom, Georgia, Ghana, Gambia, Guinea, Equatorial Guinea, Greece, Guinea-Bissau, Croatia, Ireland, Israel, India, Iraq, Iran, Iceland, Italy, Kenya, South Korea, Liberia, Luxembourg, Latvia, Libyan Arab Jamahiriya, Morocco, Myanmar, Mauritania, Mexico, Malaysia, Mozambique, Niger, Nigeria, Netherland, Norway, New Zealand, Oman, Peru, Pakistan, Poland, Portugal, Romania, Russia, Rwanda, Sudan, Sweden, Singapore, Slovenia, Slovakia, Sierra Leone, Senegal, Somalia, Suriname, Sao Tome and Principe, Syrian Arab Republic, Togo, Thailand, Tunisia, Tonga, Turkey, Tanzania, Ukraine, Uganda, USA, Uruguay, Venezuela, Serbia, Zambia, Zimbabwe and South Africa. However, only to keep the number of instances in the variable country of origin similar to the number of instance in the variable country of sampling; only instances of Austria, Belgium, Cameroon, CS (Serbia), Czech Republic, Germany, Denmark, Spain, Ethiopia, Finland, Greece, Italy, Nigeria, The Netherlands, Norway, Poland, Portugal, Sweden, Slovenia, Thailand and Yugoslavia were left ungrouped; while all other instances of country of origin with smaller sample size were group together.

Logistic regression, however, is not the best method to determine dependency between the variables. For that, we used Bayesian Network analysis. Its use helps to determine which associations occur directly between the analysed variables and which associations are secondary to other direct primary associations. In our Bayesian network analysis, risk factor, continent of origin and country of origin were identified as unconditionally associated with the infecting subtype, as illustrated by a direct arc (Figure 5). However, only the arc connecting continent of origin and HIV clade was confirmed by a high bootstrap support (74% of the replicates). On the other hand, countries of sampling and gender were found to be only secondarily associated with subtype, since there is no direct arc with subtype, rather the connection is through the risk factor, continent of origin and country of origin variables. For example, the arc that connects continent of origin to HIV clade indicates that there is a direct and unconditional dependence between these two variables; therefore, the continent of origin of the patient is an important determinant of the HIV clade. On the other hand, gender is connected to HIV clade through the risk group, which means that the unequal distribution of subtypes according to gender can be explained by the fact that there is a difference in subtype epidemic among MSM (all men) compared to heterosexual or IDU (men and women). Similar extrapolations can be made to other variables of the network (Figure 5).

### The proportion of newly diagnosed with CRF02_AG and subtype F increased significantly between 2002 and 2005

**Figure 6:**
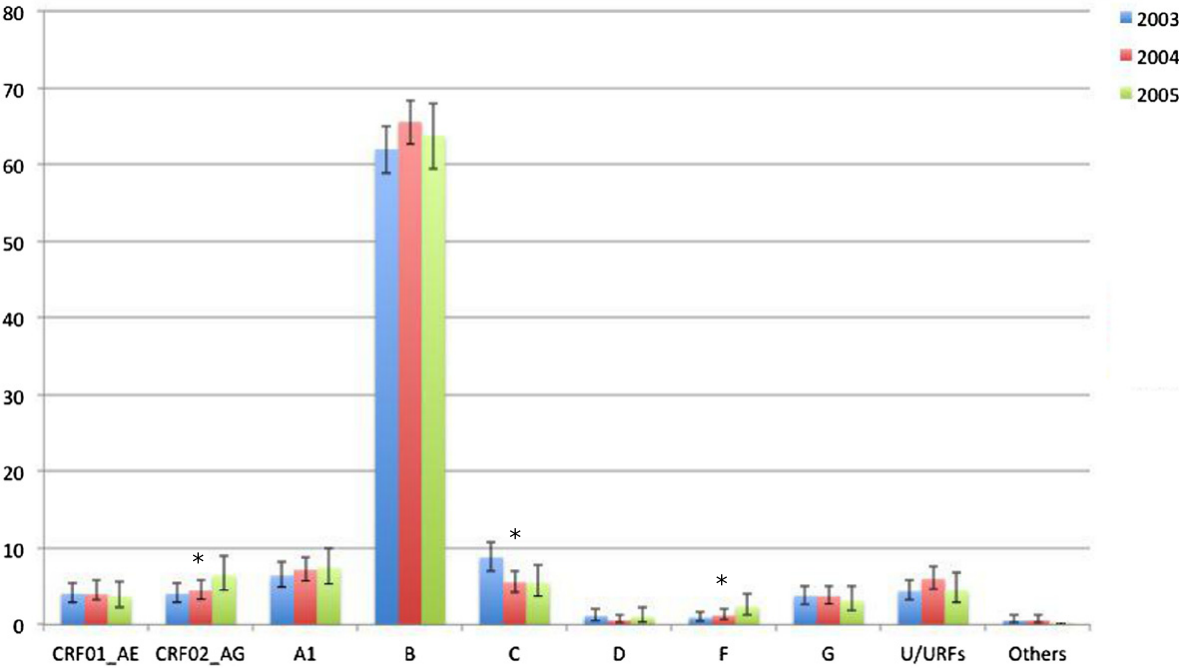
**Proportion of different HIV-1 subtypes stratified according to year of diagnosis.** In 2002, samples were collected only during the last 3 months, and therefore this year was excluded from the analysis. 909 samples were collected in 2003, 1107 in 2004 and 513 in 2005. Significance for increasing or decreasing trends was tested with the Cohran-Armitage test. Significant trends are indicated in the figure with an asterisk. p-values are described in the text.

When analysing the proportion of diagnoses with different HIV-1 subtypes stratified over the years according to date of first positive confirmatory HIV-1 test, we found that most subtypes did not show any significant trend. The exception to this rule was CRF02_AG and subtype F with a significantly increasing (p-value=0.05 and 0.03, respectively) and subtype C with a significantly decreasing trend between 2002 and 2005 (p=0.004). No significant trends were found in the proportion of diagnoses with subtype B, which increased in 2004 but then decreased in 2005 (Figure 6).

## Discussion

In this paper, we have described the HIV-1 subtype distribution and its associated socio-demographic factors in newly diagnosed patients in West-Central Europe using sequences from the SPREAD programme (http://www.esar-society.eu/). Samples were collected between 2002 and 2005 from drug-naïve patients diagnosed not earlier than 6 months before sampling, together with clinical and epidemiological information. The sampling strategy was based on representative sampling over countries and risk groups, allowing a more accurate picture than in previous studies that were either based on convenience sampling [23] or focused solely on one country [28,29]. In addition, our study is unique since it permitted to combine for the first time molecular data of a ‘continent’-scaled sample, together with demographic and behaviour information of the patients. The primary objective of the SPREAD study was to measure the extent of transmission of drug-resistant HIV, and an additional objective was to characterize the genetic diversity of the epidemic in Europe. For this second objective, we subtyped all samples from three inclusion rounds of the SPREAD programme (Sept 2002-Dec 2005), and analyzed the geographical spread and the factors associated with this subtype distribution. Although SPREAD sampling strategy was carefully designed to avoid sampling bias, we noticed that not all countries were sampled at the same density. Given the differences found in the sampling rate of newly diagnosed patients especially in small countries, we performed a weighted analysis to account for such differences. Such a strategy has, however, its own limits since diagnosis rates may differ among risk groups [30] or the proportion of infected patients that are undiagnosed may differ between countries. Although we found some significant differences with the weighted analysis, this difference was not substantial and did not result in different conclusions.

Because of its high specificity, even though at the cost of sensitivity [31,32], we only used the Rega subtyping tool in our study, and complemented it with manual analysis for unclassified sequences. Although our subtyping methodology was extremely meticulous, a limitation of any study using only one genetic region is the fact that no claims can be made with regard to the absence of recombination breakpoints outside of the sequenced genomic regions.

Similar as in the UNAIDS report for Western Europe [33], we found subtype B to be the most prevalent subtype but lower than reported before: 66.1% compared to the 85% reported by UNAIDS. In the subset of patients not only diagnosed but also originating from Europe the proportion of subtype B among newly diagnosed patients was significantly higher (79.5%) resembling the UNAIDS data. These differences are thus likely explained by the very different sampling strategies between the studies: UNAIDS used country of origin of the patient, while we used country of sampling; and UNAIDS used a convenience sample of data collected from patients diagnosed at any time and we used a representative sample of newly diagnosed patients. Subtypes A1 (6.9%), C (6.8%) and CRF02_AG (4.7%) are the other most prevalent subtypes in patients diagnosed in Europe. Again here the results are not entirely consistent with the UNAIDS report, where the most prevalent non-B subtypes in Western and Central Europe were CRF02_AG (2000–2003: 2.94%; 2004–2007: 4.50%) and subtype C (2000–2003: 2.9%; 2004–2007: 1.91%). As in our study only newly diagnosed patients were included in the sample, a major advantage of our sampling strategy is that it maps the more recent past compared to previous studies. Our findings may therefore be more relevant for the epidemic in the near future.

The information collected allowed us to make a detailed analysis of the relationship between the socio-demographic and other epidemiological indicators of the patient and the HIV subtype. Despite the increasing spread of different non-B subtypes, the continent of origin and the risk group of the patient are still good predictors of the subtype. While for now, we are still able to attribute the proportion of new diagnoses with many non-B subtypes in Europe to certain demographic groups; this might change in the future. For example, subtype G has already a well-established epidemic in Portugal even within natives and in both heterosexual and IDU risk groups. From our results, it is clear that some non-B subtypes are still being imported into Europe and remain largely limited to migrant populations, such as CRF02_AG in Belgium; while some have established themselves in the native European population, like subtype G in Portugal and subtype A1 in Greece. Similar observations can be made for the risk group analysis, where subtypes are clearly compartmentalized in different transmission groups. This suggests highly stratified epidemics occurring in each country and risk group. HIV infection is still determined by social, behavioural and demographic characteristics of the patients, and we should thus target preventive measures to specific populations.

The subtype B epidemic was firstly described in the MSM population [34], but was found in IDUs soon afterwards [35]. Interestingly, the prevalence of subtype B is still higher in the MSM risk group. This could either indicate a higher transmission potential of subtype B in MSM, or could just be a reflection of the long-term establishment of subtype B infection in this risk group, that may be more compartmentalized than IDUs. Even though some reports are consistent with the hypothesis of a biological difference between subtypes with regard to transmission rates and routes [36,37], with respect to our findings, more data are needed to exclude simple epidemiological circumstances. In this study, we find that heterosexuals are more frequently infected with non-B subtypes, and this is reflected in the higher proportion of women – and consequently probably children - infected with such strains. Finally, we find that the MSM risk group presents a recent rise of proportion of sub-subtype A1 infections (4.9%), mostly caused by an epidemic among Greek MSMs.

The proportion of new diagnoses with HIV-1 CRF02_AG and subtype F increased significantly between 2002 and 2005, while for subtype C we saw a significant decrease. No other significant time trends were found, indicating stable epidemics of most HIV-1 subtypes. However, given the short time period studied and the fact that most of the patients have an unknown date of infection, no firm conclusions should be made in this respect.

Although different subtypes of HIV-1 represent different epidemics, they have been dealt as a single epidemic in UNAIDS and ECDC reports. Herein, we present the social, behavioural and demographic determinants of HIV-1 subtype distribution in Europe. Stratifying results by subtypes allows a better understanding of changing prevalence and mobility of the virus.

## Methods

### Sample collection

Sequences included in the study were from 20 European countries - Austria (AT), Belgium (BE), Cyprus (CY), Denmark (DK), Finland (FI), Germany (DE), Greece (GR), Ireland (IE), Italy (IT), Luxembourg (LU), Netherlands (NL), Norway (NO), Poland (PL), Portugal (PT), Slovenia (SI), Spain (ES), Sweden (SE), Serbia (CS), Czech Republic (CZ), Slovakia (SK) - and Israel (IL). Samples were collected from HIV-1 infected individuals in whom infection was newly diagnosed between September 2002-December 2005, no longer than 6 months before sampling. To guarantee representativeness in each country, individuals were selected according to the national distribution of transmission risk groups and the geographical distribution of patients with new diagnoses of HIV-1 infection. The strategies used to achieve this were: in countries where more than 80% of all newly diagnosed individuals were expected to be covered by the participating centers, a random sample from all newly identified individuals was taken. In other countries, stratified sampling weighted for the proportion of newly diagnosed patients among different risk groups and among different geographical areas was performed or a consecutive number of patients up to a predefined number per geographic region were included. All recruited patients were antiretroviral drug naive at the time of sampling, and drug resistance genotyping was performed in the national reference laboratories as described before [25,26]. Details about the study design were reported previously and can be found on the website (http://www.esar-society.eu/) [25,26].

Although the sampling strategy was cautiously designed in order to representatively include different countries and risk groups, unavoidably some discrepancies in sampling numbers occurred between countries, especially to attain a representative sample in small countries. To assess the impact of such oversampling in some countries, the weighted proportion of newly diagnosed was calculated. The HIV infection rate per country (number of yearly newly diagnosed HIV-1 cases per inhabitant) was obtained for each included country (ECDC Report 2004, derived from infection rate per million) [33], and we estimated which percentage of infected patients was sampled in each country. Since the collection period was 39 months, we adjusted the number of samples to a 12 months period. Mathematically, the proportion of the HIV infected that was sampled corresponds to:

$$\mathrm{\% of infected inhabitants in country A}=\frac{\frac{\left(\text{Number}\;\text{of}\;\text{samples}\;\text{collected}\;\text{in}\;\text{country}\;A\right)\times 12\;\text{months}}{\left(\text{Population}\;\text{size}\;\text{country}\;A\right)\times 39\;\text{months}}}{\frac{\text{Rate}\;\text{per}\;\text{million}\;2004\;\text{of}\;\text{country}\;A}{1000000}}$$

Since the % of infected inhabitants sampled was variable between countries, the counts of subtypes of each country were weighted accordingly in the determination of the proportion of newly diagnosed patients with different subtypes in Europe. Although there were some significant differences, the overall conclusions of our analysis did not change. Therefore, the unweighted proportion is given, unless specified, and all weighted analyses can be found in supplementary material.

### Subtyping

#### Automatic subtyping

Sequences were subtyped using the REGA Subtyping tool version 2 (http://jose.med.kuleuven.ac.be/genotypetool/html/subtypinghiv.html) [31,38]. Detailed information about the algorithm of the REGA Subtyping tool version 2 are available on the website: http://www.bioafrica.net/subtypetool/html/.

Reports generated by the subtyping tool were individually viewed and a csv formatted file with the results was downloaded.

#### Manual subtyping

For sequences that were too complex for the REGA subtyping tool to assign it to a subtype or CRF automatically and that were therefore classified as ‘Unassigned’, a manual subtyping procedure was used. In this procedure, the sampled sequences were aligned against reference sequences of all pure subtypes and the reference sequences of the first 14 CRFs, using the reference set as described in the Los Alamos database. Although 51 CRFs have been described, CRFs 15 to 51 are not responsible for important epidemics and a BLAST search indicated none were present among our data. Therefore, we decided to leave them out from the phylogenetic analysis. The multiple alignment was generated using ClustalW [39] and manually edited with Se-Al v2.0 [40]. The sequences were then tested for evidence of recombination using the bootscan plot as implemented in Simplot v3.5.1. Phylogenetic analyses were performed with and without including CRF reference sequences in the datasets. The putative recombination pattern was confirmed by separate phylogenetic analysis in the fragments with different evolutionary history. Any genomic region was assigned to a certain HIV subtype if it clustered with reference sequences of this subtype and this clustering was supported by bootstrap values higher than 70%.

#### Statistical analysis

Potential associations between demographic and other parameters (area of transmission, ethnicity, etc.) and the distribution of B and non-B subtypes were statistically analysed. The SPREAD questionnaire included information about gender, age, risk factor, continent and country of origin, country of sampling and country where infection was obtained. The univariate analysis of association between these factors and proportion of B vs non-B, C vs non-C, A1 vs non-A1, G vs non-G, CRF01_AE vs non-CRF01AE, CRF02AG vs non-CRF02AG subtypes and URFs vs non-URFs was tested using the Chi-square test. The p-values of the chi-square test were calculated using the R package [41]. The Holm-Bonferroni method was used to check whether multiple testing could lead to a false rejection of the null hypothesis (type I error). The odds ratio (OR) and the 95% confidence interval (CI) of the OR were calculated using a small script in Microsoft Excel. A multivariate analysis was also done with stepwise logistic regression, using as start variables: a) all variables; b) only the variables that were statistically significant in the previously described univariate analysis (p≤ 0.05). Both binary and multinomial logistic regression were performed with the R package.

Bayesian networks (BN) were run for variables that were significantly associated with the distribution of HIV-1 subtype/CRF using the univariate analysis. A BN is a probabilistic graphical model that illustrates the relationships among a set of variables. These relationships – dependencies - are defined by a set of nodes that represent the variables and a set of arcs that represent direct/unconditional dependencies between two variables in the dataset. The lack of an arc between two variables represents a conditional independency, meaning that these two variables are only dependent through another variable [42]. This analysis allows to map the interdependence of the analysed parameters unveiling direct and indirect associations with the HIV-1 subtype/CRF. The best BN that models the observed correlations is determined by a scoring metric (trade-off between model complexity and accuracy), and we use a Bayesian metric that considers the most probable one as the best network (maximizing posterior probability of the model given the data). Since an exact search is computationally impossible, we use the search heuristic of simulated annealing. We then use non-parametric bootstrap resampling to assess how strongly the data support the most probable network. A bootstrap analysis with 100 replicates was then used to investigate the reproducibility of each arc of the BN. 70% bootstrap support was used as the cut-off to assign reliable arcs. To remove the bias caused by variable instances that are present in very few patients (less than 1%), we combined those instances together in a single instance called ‘Others’. This procedure was done using the preprocessing filter available in the WEKA software.

Finally, to test for time trends in subtype distribution, we used the Cochran-Armitage test as implemented in the prop.trend.test function in the stats package of the R package.

## Competing interests

The authors declare that they have no competing interests.

## Authors’ contributions

ABA, AMJW and AMV designed and implemented the analysis. ABA, JV and KT performed the analysis. ABA, DP and AMV drafted the manuscript. AMJW, JA, BA, CB, DB, RJC, BC, CDG, AG, ZG, OH, AH, TK, KK, LGK, CK, KL, ML, CN, DO, RP, MP, EP-S, J-CS, AS, DS, MS, DS and CABB contributed clinical and virological data. All authors reviewed and/or revised the manuscript and contributed to the interpretation of the results. All co-authors have read and approved the final manuscript.

## Supplementary Material

Additional file 1 **Figure S1.** Percentage of samples of the dataset sampled in each country (dark grey) (see methods for details on calculations involved) and percentage of infected inhabitants that was sampled in each country (white) as reported by the ECDC-UNAIDS in the 2004 report. AT – Austria, BE – Belgium, CY – Cyprus, DK – Denmark, FI – Finland, DE – Germany, GR – Greece, IE – Ireland, IT – Italy, LU – Luxembourg, NL – Netherlands, NO – Norway, PL – Poland, PT – Portugal, SI – Slovenia, ES – Spain, SE – Sweden, CS – Serbia, CZ – Czech Republic, SK – Slovakia, IL – Israel. Click here for file

Additional file 2 **Figure S2.** Prevalence of subtypes for the complete dataset of patients not adjusted (left bars), for the complete dataset of patients adjusted according to size of the sample with respect to the epidemic (middle bars) and for the set of patients originating from SPREAD countries (right bars). Legend presents percentage values and 95% confidence intervals for each bar. See methods for details on the procedure to adjust for sampling bias. Asterisks indicate statistically significant differences in the prevalence of a certain subtype (p<0.05) when comparing the complete dataset and the dataset including only patients originating from SPREAD countries. Click here for file

Additional file 3 **Figure S3.** Subtypes distribution by country of sampling of the patient. AT – Austria, BE – Belgium, CY - Cyprus, DK – Denmark, FI – Finland, DE – Germany, GR – Greece, IE – Ireland, IT – Italy, LU – Luxembourg, NL – Netherlands, NO – Norway, PL – Poland, PT – Portugal, SI – Slovenia, ES – Spain, SE – Sweden, CS – Serbia, CZ – Czech Republic, SK – Slovakia, IL – Israel. Click here for file

Additional file 4 **Table S1.** – Subtypes distribution by country of sampling of the patient. **Table S2.** – Subtypes distribution by country of origin of the patient. AO – Angola, AT – Austria, BE – Belgium, BI – Burundi, BR – Brazil, CG – Congo, CM – Cameroon, CS – Serbia, CV – Cape Verde, CY – Cyprus, CZ – Czech Republic, DE – Germany, DK – Denmark, ES – Spain, ET – Ethiopia, FI – Finland, GR – Greece, IE – Ireland, IT – Italy, KE – Kenya, NG – Nigeria, NL – The Nederlands, NO – Norway, PL – Poland, PT – Portugal, RU – Russian Federation, SE – Sweden, SI – Slovenia, SK – Slovakia, TH – Thailand, UA – Ukraine,YU – Yugoslavia. **Table S3.** – Goodness of fit for the logistic model, Odds ratio with Confidence Interval and p-values for the association between HIV-1 subtypes prevalence and demographic parameters. Associations were calculated using binomial logistic regression (see methods for details). Sub-Saharan Africa - SSA, South and South-East Asia –SS EA. Eastern Europe and Central Asia - EE CA. Western Europe – WE. **Table S4.** – Goodness of fit for the logistic model, Odds ratio with Confidence Interval and p-values for the association between HIV-1 subtypes prevalence and demographic parameters. Associations were calculated using multinomial logistic regression (see methods for details). **Table S5.** – List of countries included in each continent region. Click here for file

Additional file 5 **Figure S4.** Subtypes distribution by country of origin of the patient. Left: DE – Germany, GR – Greece, PT – Portugal, PL – Poland, ES – Spain, IT – Italy, BE – Belgium, CZ – Czech Republic, DK – Denmark, SE – Sweden, AT – Austria, SI – Slovenia, FI – Finland, NL – The Nederlands, NO – Norway. Right: ET – Ethiopia, CM – Cameroon, YU – Yugoslavia, TH – Thailand, NG – Nigeria, CS – Serbia, IE – Ireland, AO – Angola, CY – Cyprus, UA – Ukraine, RU – Russian Federation, SK – Slovakia, CG – Congo, KE – Kenya, BI – Burundi, BR – Brazil, CV – Cape Verde. Click here for file
