# Supplementary figures and images for: HIV-1 subtype distribution and its demographic determinants in newly diagnosed patients in Europe suggest highly compartmentalized epidemics
Source: Retrovirology. 2013 Jan 14;10:7. doi: 10.1186/1742-4690-10-7 (PMC3564855; doi:10.1186/1742-4690-10-7)

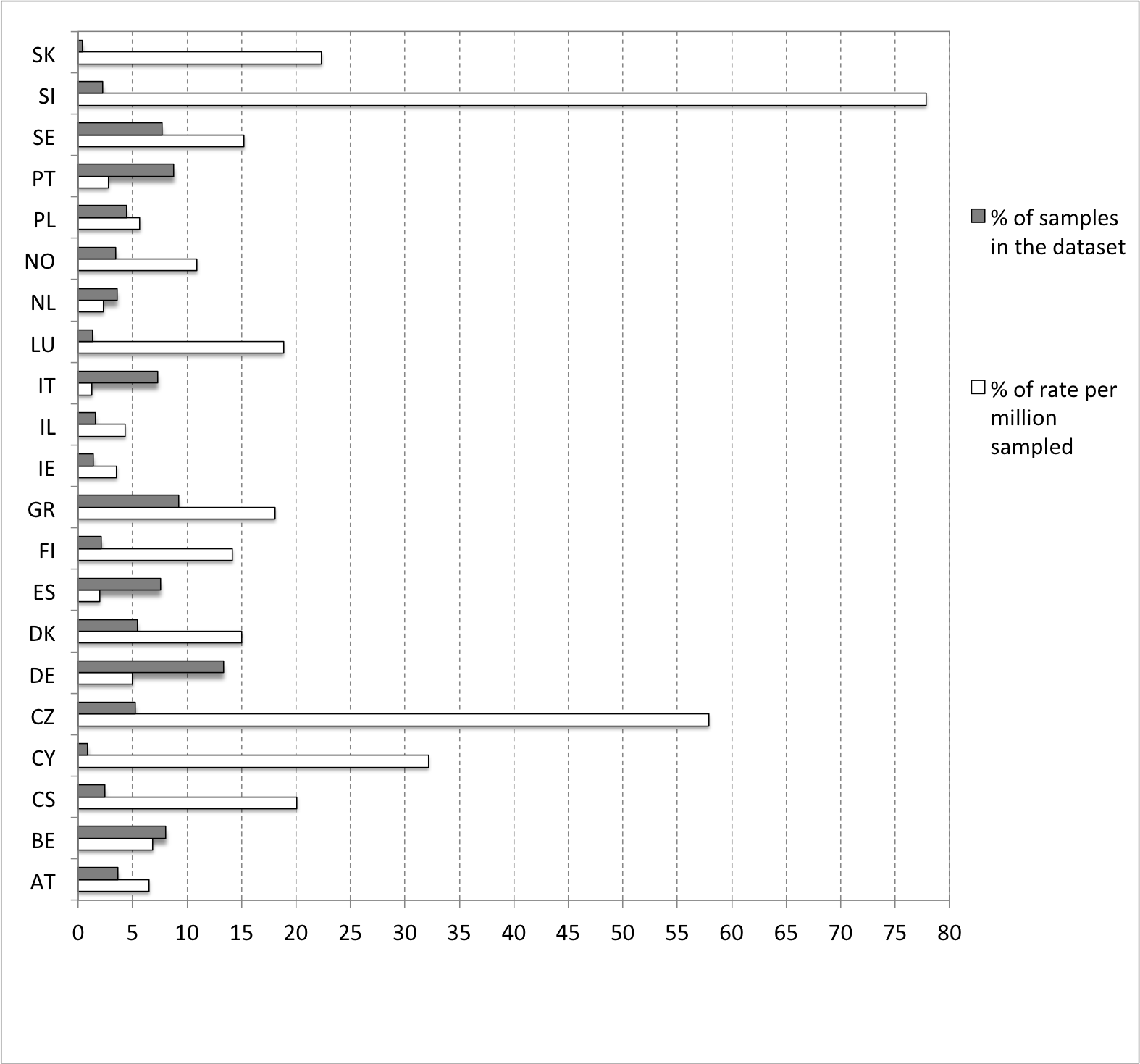

Supplement: Additional file 1 — Figure S1. Percentage of samples of the dataset sampled in each country (dark grey) (see methods for details on calculations involved) and percentage of infected inhabitants that was sampled in each country (white) as reported by the ECDC-UNAIDS in the 2004 report. AT – Austria, BE – Belgium, CY – Cyprus, DK – Denmark, FI – Finland, DE – Germany, GR – Greece, IE – Ireland, IT – Italy, LU – Luxembourg, NL – Netherlands, NO – Norway, PL – Poland, PT – Portugal, SI – Slovenia, ES – Spain, SE – Sweden, CS – Serbia, CZ – Czech Republic, SK – Slovakia, IL – Israel. [file 1742-4690-10-7-S1.png]

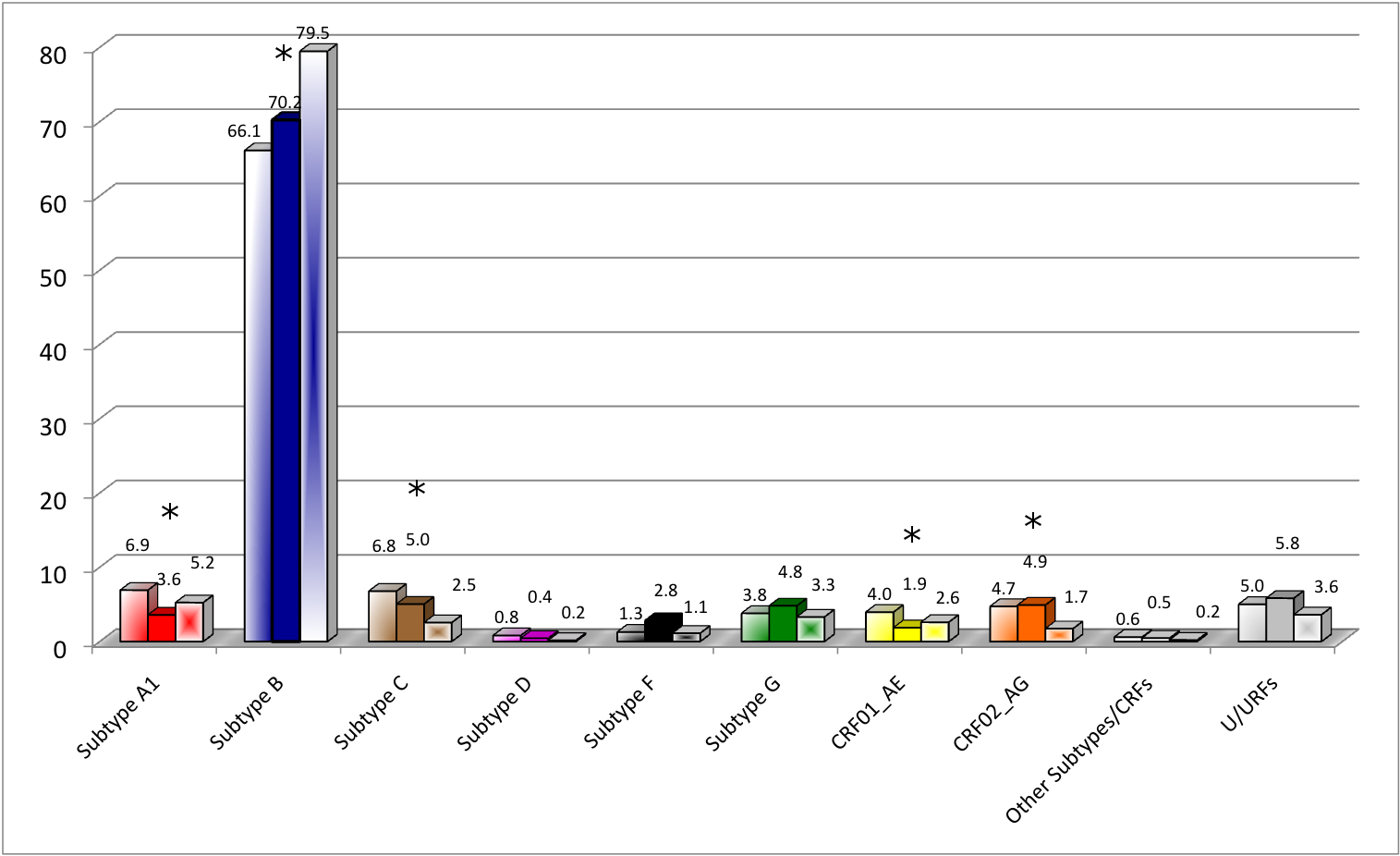

Supplement: Additional file 2 — Figure S2. Prevalence of subtypes for the complete dataset of patients not adjusted (left bars), for the complete dataset of patients adjusted according to size of the sample with respect to the epidemic (middle bars) and for the set of patients originating from SPREAD countries (right bars). Legend presents percentage values and 95% confidence intervals for each bar. See methods for details on the procedure to adjust for sampling bias. Asterisks indicate statistically significant differences in the prevalence of a certain subtype (p<0.05) when comparing the complete dataset and the dataset including only patients originating from SPREAD countries. [file 1742-4690-10-7-S2.png]

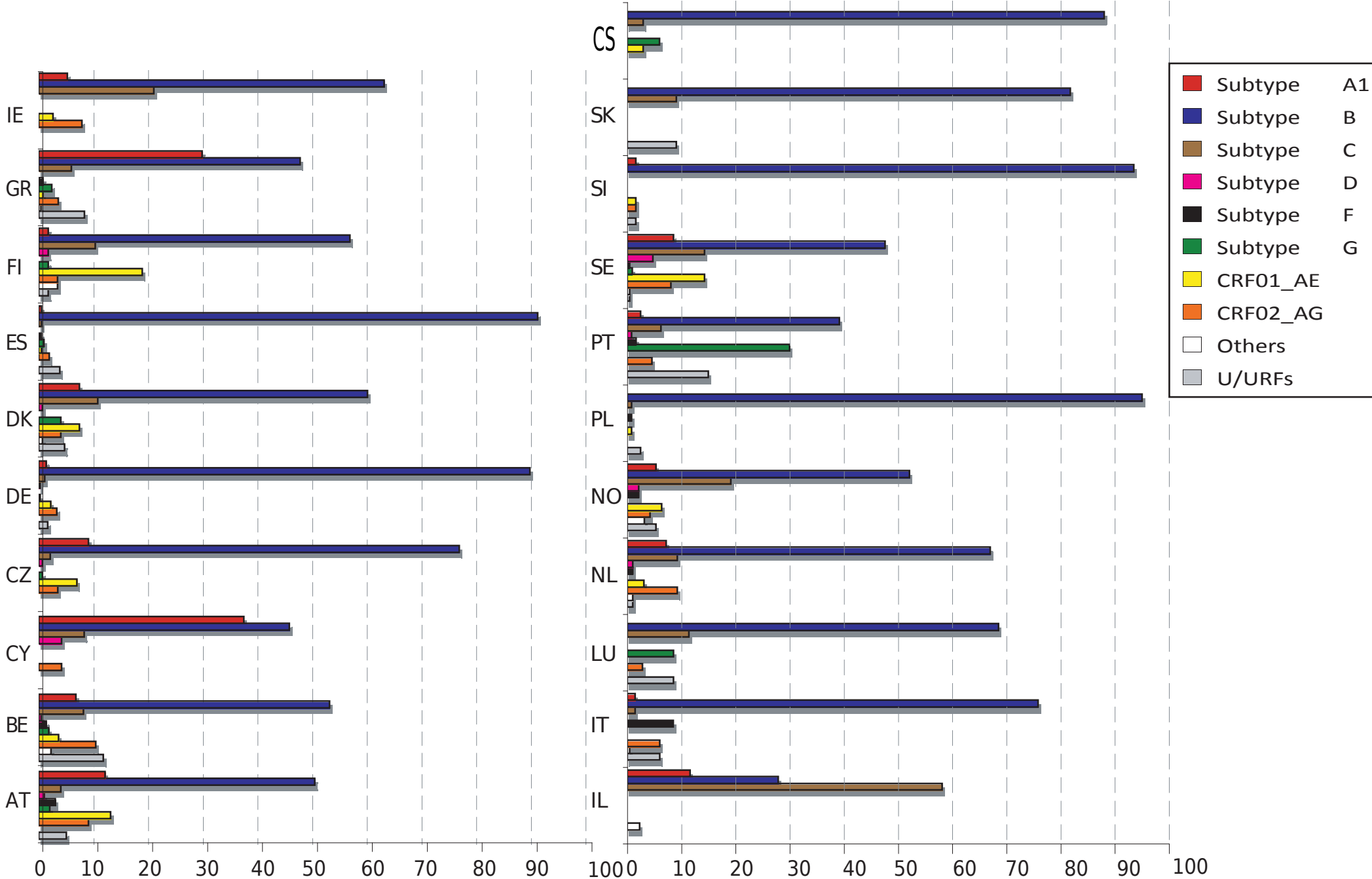

Supplement: Additional file 3 — Figure S3. Subtypes distribution by country of sampling of the patient. AT – Austria, BE – Belgium, CY - Cyprus, DK – Denmark, FI – Finland, DE – Germany, GR – Greece, IE – Ireland, IT – Italy, LU – Luxembourg, NL – Netherlands, NO – Norway, PL – Poland, PT – Portugal, SI – Slovenia, ES – Spain, SE – Sweden, CS – Serbia, CZ – Czech Republic, SK – Slovakia, IL – Israel. [file 1742-4690-10-7-S3.pdf]

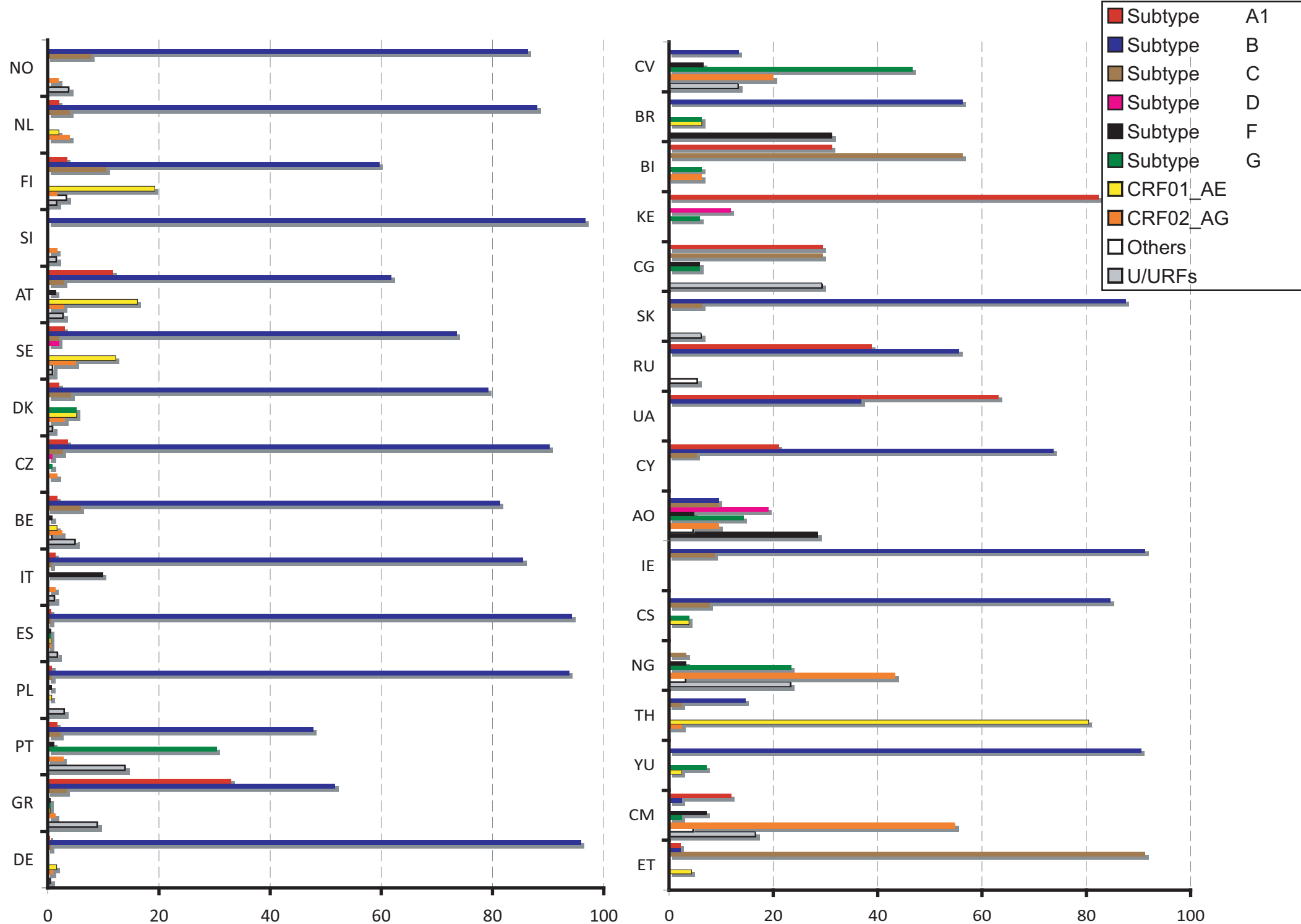

Supplement: Additional file 5 — Figure S4. Subtypes distribution by country of origin of the patient. Left: DE – Germany, GR – Greece, PT – Portugal, PL – Poland, ES – Spain, IT – Italy, BE – Belgium, CZ – Czech Republic, DK – Denmark, SE – Sweden, AT – Austria, SI – Slovenia, FI – Finland, NL – The Nederlands, NO – Norway. Right: ET – Ethiopia, CM – Cameroon, YU – Yugoslavia, TH – Thailand, NG – Nigeria, CS – Serbia, IE – Ireland, AO – Angola, CY – Cyprus, UA – Ukraine, RU – Russian Federation, SK – Slovakia, CG – Congo, KE – Kenya, BI – Burundi, BR – Brazil, CV – Cape Verde. [file 1742-4690-10-7-S5.pdf]
