# Supplementary material for: HIV-1 subtype distribution and its demographic determinants in newly diagnosed patients in Europe suggest highly compartmentalized epidemics
Source: Retrovirology. 2013 Jan 14;10:7. doi: 10.1186/1742-4690-10-7 (PMC3564855; doi:10.1186/1742-4690-10-7)
Supplement: Additional file 4 — Table S1. – Subtypes distribution by country of sampling of the patient. Table S2. – Subtypes distribution by country of origin of the patient. AO – Angola, AT – Austria, BE – Belgium, BI – Burundi, BR – Brazil, CG – Congo, CM – Cameroon, CS – Serbia, CV – Cape Verde, CY – Cyprus, CZ – Czech Republic, DE – Germany, DK – Denmark, ES – Spain, ET – Ethiopia, FI – Finland, GR – Greece, IE – Ireland, IT – Italy, KE – Kenya, NG – Nigeria, NL – The Nederlands, NO – Norway, PL – Poland, PT – Portugal, RU – Russian Federation, SE – Sweden, SI – Slovenia, SK – Slovakia, TH – Thailand, UA – Ukraine,YU – Yugoslavia. Table S3. – Goodness of fit for the logistic model, Odds ratio with Confidence Interval and p-values for the association between HIV-1 subtypes prevalence and demographic parameters. Associations were calculated using binomial logistic regression (see methods for details). Sub-Saharan Africa - SSA, South and South-East Asia –SS EA. Eastern Europe and Central Asia - EE CA. Western Europe – WE. Table S4. – Goodness of fit for the logistic model, Odds ratio with Confidence Interval and p-values for the association between HIV-1 subtypes prevalence and demographic parameters. Associations were calculated using multinomial logistic regression (see methods for details). Table S5. – List of countries included in each continent region. [file 1742-4690-10-7-S4.docx]

|  |  | AT | BE | CS | CY | CZ | DE | DK | ES | FI | GR | IE | IL | IT | LU | NL | NO | PL | PT | SE | SI | SK |
| --- | --- | --- | --- | --- | --- | --- | --- | --- | --- | --- | --- | --- | --- | --- | --- | --- | --- | --- | --- | --- | --- | --- |
| Subtype A1 | N  % | 12  12.1 | 15  6.8 | 0  0.0 | 9  37.5 | 13  9.1 | 5  1.4 | 11  7.4 | 1  0.5 | 1  1.7 | 75  29.9 | 2  5.3 | 5  11.6 | 3  1.5 | 0  0.0 | 7  7.2 | 5  5.3 | 0  0.0 | 6  2.5 | 18  8.6 | 1  1.6 | 0  0.0 |
| Subtype B | N  % | 50  50.5 | 117  53.2 | 59  88.1 | 11  45.8 | 110  76.9 | 327  89.8 | 89  60.1 | 188  91.3 | 33  56.9 | 120  47.8 | 24  63.2 | 12  27.9 | 151  75.9 | 24  68.6 | 65  67.0 | 49  52.1 | 115  95.0 | 94  39.2 | 100  47.6 | 58  93.6 | 9  88.1 |
| Subtype C | N  % | 4  4.0 | 18  8.2 | 2  3.0 | 2  8.3 | 3  2.1 | 4  1.1 | 16  10.8 | 1  0.5 | 6  10.3 | 15  6.0 | 8  21.1 | 25  58.1 | 3  1.5 | 4  11.4 | 9  9.3 | 18  19.2 | 1  0.8 | 15  6.3 | 30  14.3 | 0  0.0 | 1  3.0 |
| Subtype D | N  % | 1  1.0 | 1  0.5 | 0  0.0 | 1  4.2 | 1  0.7 | 1  0.3 | 1  0.7 | 0  0.0 | 1  1.7 | 0  0.0 | 0  0.0 | 0  0.0 | 0  0.0 | 0  0.0 | 1  1.0 | 2  2.1 | 0  0.0 | 2  0.8 | 10  4.8 | 0  0.0 | 0  0.0 |
| Subtype F | N  % | 3  3.0 | 3  1.4 | 0  0.0 | 0  0.0 | 0  0.0 | 0  0.0 | 0  0.0 | 1  0.5 | 0  0.0 | 2  0.8 | 0  0.0 | 0  0.0 | 17  8.5 | 0  0.0 | 1  1.0 | 2  2.1 | 1  0.8 | 4  1.7 | 1  0.5 | 0  0.0 | 0  0.0 |
| Subtype G | N  % | 2  2.0 | 4  1.8 | 4  6.0 | 0  0.0 | 1  0.7 | 1  0.3 | 6  4.1 | 2  1.0 | 1  1.7 | 6  2.4 | 0  0.0 | 0  0.0 | 0  0.0 | 3  8.6 | 0  0.0 | 0  0.0 | 0  0.0 | 72  30.0 | 2  1.0 | 0  0.0 | 0  6.0 |
| CRF01_AE | N  % | 13  13.1 | 8  3.6 | 2  3.0 | 0  0.0 | 10  7.0 | 8  2.2 | 11  7.4 | 1  0.5 | 11  19.0 | 2  0.8 | 1  2.6 | 0  0.0 | 0  0.0 | 0  0.0 | 3  3.1 | 6  6.4 | 1  0.8 | 0  0.0 | 30  14.3 | 1  1.6 | 0  3.0 |
| CRF02_AG | N  % | 9  9.1 | 23  10.5 | 0  0.0 | 1  4.2 | 5  3.5 | 12  3.3 | 6  4.1 | 4  1.9 | 2  3.5 | 9  3.6 | 3  7.9 | 0  0.0 | 12  6.0 | 1  2.9 | 9  9.3 | 4  4.3 | 0  0.0 | 11  4.6 | 17  8.1 | 1  1.6 | 0  0.0 |
| Others | N  % | 0  0.0 | 5  2.3 | 0  0.0 | 0  0.0 | 0  0.0 | 0  0.0 | 1  0.7 | 0  0.0 | 2  3.5 | 1  0.4 | 0  0.0 | 1  2.3 | 1  0.5 | 0  0.0 | 1  1.0 | 3  3.2 | 0  0.0 | 0  0.0 | 1  0.5 | 0  0.0 | 0  0.0 |
| U/URFs | N  % | 5  5.1 | 26  11.8 | 0  0.0 | 0  0.0 | 0  0.0 | 6  1.7 | 7  4.7 | 8  3.9 | 1  1.7 | 21  8.4 | 0  0.0 | 0  0.0 | 12  6.0 | 3  8.6 | 1  1.0 | 5  5.3 | 3  2.5 | 36  15.0 | 1  0.5 | 1  1.6 | 1  0.0 |
| Total |  | 99 | 220 | 67 | 24 | 143 | 364 | 148 | 206 | 58 | 251 | 38 | 43 | 199 | 35 | 97 | 94 | 121 | 240 | 210 | 62 | 11 |

NOTE: Detail may not sum to totals because of rounding.

**Supplementary Table 1** – Subtypes distribution by country of sampling of the patient.

AT – Austria, BE – Belgium, CY - Cyprus, DK – Denmark, FI – Finland, DE – Germany, GR – Greece, IE – Ireland, IT – Italy, LU – Luxembourg, NL – Netherlands, NO – Norway, PL – Poland, PT – Portugal, SI – Slovenia, ES – Spain, SE – Sweden, CS – Serbia, CZ – Czech Republic, SK – Slovakia, IL – Israel.

|  | A1 | B | C | D | F | G | CRF01  AE | CRF02  AG | Other Subtypes/CRFs | U/  URFs |
| --- | --- | --- | --- | --- | --- | --- | --- | --- | --- | --- |
| AO | 0.00 | 9.52 | 9.52 | 19.05 | 4.76 | 14.29 | 0.00 | 9.52 | 4.76 | 28.57 |
| AT | 11.76 | 61.76 | 2.94 | 0.00 | 1.47 | 0.00 | 16.18 | 2.94 | 0.00 | 2.94 |
| BE | 1.69 | 81.36 | 5.93 | 0.00 | 0.85 | 0.00 | 1.69 | 2.54 | 0.85 | 5.08 |
| BI | 31.25 | 0.00 | 56.25 | 0.00 | 0.00 | 6.25 | 0.00 | 6.25 | 0.00 | 0.00 |
| BR | 0.00 | 56.25 | 0.00 | 0.00 | 0.00 | 6.25 | 6.25 | 0.00 | 0.00 | 31.25 |
| CG | 29.41 | 0.00 | 29.41 | 0.00 | 5.88 | 5.88 | 0.00 | 0.00 | 0.00 | 29.41 |
| CM | 11.90 | 2.38 | 0.00 | 0.00 | 7.14 | 2.38 | 0.00 | 54.76 | 4.76 | 16.67 |
| CS | 0.00 | 84.62 | 7.69 | 0.00 | 0.00 | 3.85 | 3.85 | 0.00 | 0.00 | 0.00 |
| CV | 0.00 | 13.33 | 0.00 | 0.00 | 6.67 | 46.67 | 0.00 | 20.00 | 0.00 | 13.33 |
| CY | 21.05 | 73.68 | 5.26 | 0.00 | 0.00 | 0.00 | 0.00 | 0.00 | 0.00 | 0.00 |
| CZ | 3.57 | 90.18 | 2.68 | 0.89 | 0.00 | 0.89 | 0.00 | 1.79 | 0.00 | 0.00 |
| DE | 0.32 | 95.86 | 0.64 | 0.00 | 0.00 | 0.00 | 1.59 | 0.96 | 0.00 | 0.64 |
| DK | 2.08 | 79.17 | 4.17 | 0.00 | 0.00 | 5.21 | 5.21 | 3.13 | 0.00 | 1.04 |
| ES | 0.64 | 94.23 | 0.64 | 0.00 | 0.64 | 0.64 | 0.64 | 0.64 | 0.00 | 1.92 |
| ET | 2.17 | 2.17 | 91.30 | 0.00 | 0.00 | 0.00 | 4.35 | 0.00 | 0.00 | 0.00 |
| FI | 3.51 | 59.65 | 10.53 | 0.00 | 0.00 | 0.00 | 19.30 | 1.75 | 3.51 | 1.75 |
| GR | 33.01 | 51.67 | 3.35 | 0.00 | 0.48 | 0.48 | 0.48 | 1.44 | 0.00 | 9.09 |
| IE | 0.00 | 91.30 | 8.70 | 0.00 | 0.00 | 0.00 | 0.00 | 0.00 | 0.00 | 0.00 |
| IT | 1.32 | 85.43 | 0.66 | 0.00 | 9.93 | 0.00 | 0.00 | 1.32 | 0.00 | 1.32 |
| KE | 82.35 | 0.00 | 0.00 | 11.76 | 0.00 | 5.88 | 0.00 | 0.00 | 0.00 | 0.00 |
| NG | 0.00 | 0.00 | 3.33 | 0.00 | 3.33 | 23.33 | 0.00 | 43.33 | 3.33 | 23.33 |
| NL | 2.00 | 88.00 | 4.00 | 0.00 | 0.00 | 0.00 | 2.00 | 4.00 | 0.00 | 0.00 |
| NO | 0.00 | 86.27 | 7.84 | 0.00 | 0.00 | 0.00 | 0.00 | 1.96 | 0.00 | 3.92 |
| PL | 0.78 | 93.80 | 0.78 | 0.00 | 0.78 | 0.00 | 0.78 | 0.00 | 0.00 | 3.10 |
| PT | 1.69 | 47.75 | 2.25 | 0.00 | 1.12 | 30.34 | 0.00 | 2.81 | 0.00 | 14.04 |
| RU | 38.89 | 55.56 | 0.00 | 0.00 | 0.00 | 0.00 | 0.00 | 0.00 | 5.56 | 0.00 |
| SE | 3.06 | 73.47 | 2.04 | 2.04 | 0.00 | 0.00 | 12.24 | 5.10 | 1.02 | 1.02 |
| SI | 0.00 | 96.61 | 0.00 | 0.00 | 0.00 | 0.00 | 0.00 | 1.69 | 0.00 | 1.69 |
| SK | 0.00 | 87.50 | 6.25 | 0.00 | 0.00 | 0.00 | 0.00 | 0.00 | 0.00 | 6.25 |
| TH | 0.00 | 14.63 | 2.44 | 0.00 | 0.00 | 0.00 | 80.49 | 2.44 | 0.00 | 0.00 |
| UA | 63.16 | 36.84 | 0.00 | 0.00 | 0.00 | 0.00 | 0.00 | 0.00 | 0.00 | 0.00 |
| YU | 0.00 | 90.48 | 0.00 | 0.00 | 0.00 | 7.14 | 2.38 | 0.00 | 0.00 | 0.00 |

**Supplementary Table 2** – Subtypes distribution by country of origin of the patient.

AO – Angola, AT – Austria, BE – Belgium, BI – Burundi, BR – Brazil, CG – Congo, CM – Cameroon, CS – Serbia, CV – Cape Verde, CY – Cyprus, CZ – Czech Republic, DE – Germany, DK – Denmark, ES – Spain, ET – Ethiopia, FI – Finland, GR – Greece, IE – Ireland, IT – Italy, KE – Kenya, NG – Nigeria, NL – The Nederlands, NO – Norway, PL – Poland, PT – Portugal, RU – Russian Federation, SE – Sweden, SI – Slovenia, SK – Slovakia, TH – Thailand, UA – Ukraine,YU – Yugoslavia.

| **Subtype/CRF** | **Goodness**  **of fit** | **Variable group** | **Variables** | **Association** | **OR** | **OR CI** | **p-value** |
| --- | --- | --- | --- | --- | --- | --- | --- |
| **CRF01_AE** | AUC=0.89 | Country of origin | Thailand | + | 80.1 | 20.5-318.0 | 3.3e^-10^ |
|  | Delta=0.02 |  | Austria | + | 16.4 | 7.6-35.5 | 1.2e^-12^ |
|  | AIC=535.2 |  | Sweden | + | 6.3 | 2.2-18.0 | 5.3e^-04^ |
|  | p=2.1e-80 |  | Finland | + | 20.6 | 9.4-45.5 | 5.7e^-14^ |
|  |  | Risk group | Homo-bisexuals | - | 0.24 | 0.1-0.6 | 0.001 |
|  |  | Continent of origin | WE | - | 0.4 | 0.2-0.7 | 1.6e^-03^ |
|  |  |  | EE CA | - | 0.08 | 0.02-0.36 | 7.7e^-04^ |
| **CRF02_AG** | AUC=0.87 | Continent of origin | SS EA | + | 4.6 | 1.2-18.1 | 0.03 |
|  | Delta=0.03 | Country of origin | Nigeria | + | 10.5 | 4.65-23.6 | 1.45e^-08^ |
|  | AIC=740.3 |  | Ghana | + | 15.1 | 5.08-44.8 | 1.01e^-06^ |
|  | p =5.1e-63 |  | Cote D’Ivoire | + | 11.1 | 3.5-35.4 | 4.3e^-05^ |
|  |  |  | Cameroon | + | 17.7 | 9.7-35.9 | 1.7e^-15^ |
|  |  |  | Liberia | + | 10.7 | 2.2-51.5 | 3.1e^-03^ |
|  |  |  | Equatorial Guinea | + | 26.5 | 2.3-303.5 | 8.4e^-03^ |
|  |  |  | Guinea | + | 28.4 | 5.0-162.9 | 1.7e^-04^ |
|  |  |  | Togo | + | 15.1 | 2.9-79.2 | 1.3e^-03^ |
|  |  |  | Sierra Leone | + | 6.2 | 5.9-648.2 | 5.9e^-04^ |
|  |  | Risk group | Homo-bisexuals | - | 0.41 | 0.22-0.78 | 6.4e^-03^ |
| **Sub-subtype A1** | AUC=0.88 | Continent of origin | SSA | + | 3.4 | 2.0-5.7 | 4.0e^-06^ |
|  | Delta=0.04 | Country of sampling | Cyprus | + | 20.3 | 7.4-55.5 | 4.8e^-09^ |
|  | AIC=960.8 | Country of origin | Greece | + | 30.6 | 19.3-48.8 | <2e^-16^ |
|  | p=2.4e-88 |  | Russian Federation | + | 42.2 | 15.1-117.7 | 8.5e^-13^ |
|  |  |  | Ukraine | + | 75.6 | 27.8-205.5 | <2e^-16^ |
|  |  |  | Georgia | + | 78.3 | 5.5-1104.5 | 0.001 |
|  |  |  | Kenya | + | 94.7 | 25.1-3579.3 | 1.99e^-11^ |
|  |  |  | Tanzania | + | 9.4 | 2.8-32.2 | 0.0003 |
|  |  |  | Democratic Republic of Congo | + | 7.4 | 2.2-25.1 | 0.001 |
|  |  |  | Uganda | + | 8.7 | 2.7-28.3 | 0.0003 |
|  |  | Risk Group | Hetero | + | 2.1 | 1.4-3.2 | 0.0009 |
| **Subtype B** | AUC=0.88 | Continent of origin | SSA | - | 1.6^e-08^ | 0.0-0.14 | 1.5e^-15^ |
|  | Delta=0.13 | Country of sampling | Spain | + | 2.7 | 1.3-5.8 | 0.01 |
|  | AIC=2243.3 |  | Greece | - | 0.4 | 0.16-0.96 | 0.04 |
|  | p=1.6e-286 |  | Portugal | - | 0.46 | 0.33-0.65 | 8.62^e-06^ |
|  |  | Country of origin | Germany | + | 2.4 | 1.4-3.9 | 0.001 |
|  |  |  | Slovenia | + | 5.7 | 1.3-24.3 | 0.02 |
|  |  |  | Italy | + | 2.7 | 1.6-4.5 | 9.94e^-05^ |
|  |  |  | Czech Rep. | + | 3.4 | 1.8-6.6 | 0.0003 |
|  |  |  | Thailand | - | 0.086 | 0.03-0.22 | 3.98e^-07^ |
|  |  |  | Cameroon | - | 0.088 | 0.01-0.79 | 0.03 |
|  |  | Risk group | Homo-bi | + | 3.5 | 2.6-4.8 | 1.7e^-15^ |
|  |  |  | Hetero | - | 0.5 | 0.4-0.7 | 1.60e-^06^ |
| **Subtype C** | AUC=0.90 | Continent of origin | EE CA | - | 0.4 | 0.2-0.9 | 0.03 |
|  | Delta=0.04 | Country of origin | Ethiopia | + | 71.8 | 22.7-227.0 | 3.38e^-13^ |
|  | AIC=807.5 |  | Somalia | + | 20.2 | 6.1-66.8 | 8.30e^-07^ |
|  | p=1.2e-110 |  | Burundi | + | 15.5 | 5.6-43.1 | 1.52e^-07^ |
|  |  |  | India | + | 43.5 | 7.4-255.5 | 3.00e^-05^ |
|  |  |  | Zimbabwe | + | 50.8 | 10.1-255.8 | 1.88e^-06^ |
|  |  |  | Tanzania | + | 9.3 | 2.9-30.6 | 0.0002 |
|  |  |  | Zambia | + | 92.8 | 11.1-777.9 | 2.94e^-05^ |
|  |  |  | Mozambique | + | 15.9 | 3.7-69.2 | 0.0002 |
|  |  |  | Homo | - | 0.14 | 0.05-0.36 | 0.0007 |
| **Subtype G** | AUC=0.90 | Country of sampling | Portugal | + | 14.9 | 7.2-31.0 | 4.68e^-13^ |
|  | Delta=0.03 | Country of origin | Nigeria | + | 17.1 | 6.7-43.7 | 3.01e^-09^ |
|  | AIC=574.1 |  | Portugal | + | 2.4 | 1.2-4.9 | 0.0148 |
|  | p=2.1e-68 | Risk group | Homo | - | 0.097 | 0.03-0.27 | 8.28e^-06^ |

**Supplementary table 3** – Goodness of fit for the logistic model, Odds ratio with Confidence Interval and p-values for the association between HIV-1 subtypes prevalence and demographic parameters. Associations were calculated using binomial logistic regression (see methods for details).

Sub-Saharan Africa - SSA,

South and South-East Asia –SS EA

Eastern Europe and Central Asia - EE CA

Western Europe – WE

| **Goodness**  **of fit** | **Variable group** | **Variables** | **Association** | **OR** | **OR CI** | **p-value** |
| --- | --- | --- | --- | --- | --- | --- |
| AUC=0.95 | Sex | Female | + | 4.6 | 1.9-11.0 | 0.000584 |
| AIC=437.02 | Risk Group | Hetero | - | 0.05 | 0.02-0.1 | 2.8e-11 |
| p=2.8e-52 |  | IDUs | - | 0.2 | 0.06-0.8 | 0.03 |
|  | Country | Austria |  |  |  | 0.07 |
|  |  | Spain |  |  |  | 0.1 |
|  |  | Finland |  |  |  | 0.06 |
|  |  | Greece |  |  |  | 0.1 |
|  |  | Norway |  |  |  | 0.1 |
|  |  | Serbia |  |  |  | 0.07 |
|  | Continent of Origin | East Asia and Pacific | - | 0.01 | 0.0002-0.5 | 0.02 |
|  |  | Eastern Europe and Central Asia | + | 6.3 | 2.6-1570 | 0.01 |
|  |  | South and South-East Asia | - | 0.0097 | 0.003-0.03 | 9.2e-15 |
|  |  | Sub-Saharan Africa | + | 9.9 | 2.3-44.4 | 0.002 |

**Supplementary Table 4** – Goodness of fit for the logistic model, Odds ratio with Confidence Interval and p-values for the association between HIV-1 subtypes prevalence and demographic parameters. Associations were calculated using multinomial logistic regression (see methods for details).

| Continent of origin | Countries included |
| --- | --- |
| Sub-Saharan Africa | Angola, Benin, Botswana, Burkina Faso, Burundi, Cameroon, Central African Republic, Chad, Comoros, Congo, Cote D’Ivoire, Democratic Republic of Congo, Djibouti, Equatorial Guinea, Eritrea, Ethiopia, Gabon, Gambia, Ghana, Guinea, Guinea-Bissau, Kenya, Lesotho, Liberia, Madagascar, Malawi, Mali, Mauritania, Mauritius, Mozambique, Namibia, Niger, Nigeria, Rwanda, Senegal, Sierra Leone, Somalia, South Africa, Swaziland, Togo,  Uganda, United Republic of Tanzania, Zambia, Zimbabwe. |
| East Asia | China, Dem People Rep. of Korea, Japan, Mongolia, Republic of Korea. |
| Oceania | Australia, Fiji, New Zealand, Papua New Guinea. |
| South and South-East Asia | Afghanistan, Bangladesh, Bhutan, Brunei Darussalam, Cambodja, India, Indonesia, Iran, Lao People’s Dem. Rep., Malaysia, Maldives, Myanmar, Nepal, Pakistan, Philippines, Singapore, Sri Lanka, Thailand, Timor-Leste, Vietnam. |
| Eastern Europe and Central Asia | Armenia, Azerbaijan, Belarus, Bosnia and  Herzegovina, Bulgaria, Croatia, Czech Republic, Estonia, Georgia, Kazakhstan, Kyrgyzstan, Latvia, Lithuania, Former Yugoslav Republic of Macedonia, Montenegro, Poland, Republic of Moldova, Romania, Russian Federation, Serbia, Slovakia, Slovenia, Tajikistan, Turkmenistan, Ukraine, Uzbekistan. |
| Western Europe | Albania, Austria, Belgium, Denmark, Finland, France,  Germany, Greece, Hungary, Iceland, Ireland, Israel, Italy, Luxembourg, Malta,Netherlands, Norway, Portugal, Spain, Sweden, Switzerland, United Kingdom of Great Britain and Northern Ireland. |
| North Africa and Middle East | Algeria, Bahrain, Cyprus, Egypt, Iraq, Jordan, Kuwait, Lebanon, Libyan Arab Jamahiriya, Morocco, Oman, Qatar, Saudi Arabia, Sudan, Syrian Arab Republic, Tunisia, Turkey, United Arab Emirates, Yemen. |
| North America | Canada, United States of America. |
| Caribbean | Bahamas, Barbados, Cuba, Dominican Republic, Haiti, Jamaica, Netherland Antilles, Trinidad and Tobago. |
| Latin America | Argentina, Belize, Bolivia, Brazil, Chile, Colombia, Costa Rica, Ecuador, El Salvador, Guatemala, Guyana, Honduras, Mexico, Nicaragua, Panama, Paraguay, Peru, Suriname, Uruguay, Venezuela. |

**Supplementary table 5** – List of countries included in each continent region.
